# Minimum Clinically Important Difference of Gross Motor Function and Gait Endurance in Children with Motor Impairment: A Comparison of Distribution-Based Approaches

**DOI:** 10.1155/2020/2794036

**Published:** 2020-05-15

**Authors:** Fabio Alexander Storm, Maurizio Petrarca, Elena Beretta, Sandra Strazzer, Luigi Piccinini, Cristina Maghini, Daniele Panzeri, Claudio Corbetta, Roberta Morganti, Gianluigi Reni, Enrico Castelli, Flaminia Frascarelli, Alessandra Colazza, Giampietro Cordone, Emilia Biffi

**Affiliations:** ^1^Scientific Institute, IRCCS “E. Medea”, Bioengineering Laboratory, Bosisio Parini, Lecco, Italy; ^2^Bambino Gesù Children's Hospital, Neurosciences and Neurorehabilitation, Rome, Italy; ^3^Scientific Institute, IRCCS “E. Medea”, Acquired Brain Injury Unit, Bosisio Parini, Lecco, Italy; ^4^Scientific Institute, IRCCS “E. Medea”, Functional Rehabilitation Unit, Bosisio Parini, Lecco, Italy

## Abstract

**Objective:**

The minimum clinically important difference (MCID) is a standard way of measuring clinical relevance. The objective of this work was to establish the MCID for the 6-minute walking test (6minWT) and the Gross Motor Function Measure (GMFM-88) in pediatric gait disorders.

**Methods:**

A cohort, pretest-posttest study was conducted in a hospitalized care setting. A total of 182 patients with acquired brain injury (ABI) or cerebral palsy (CP) performed 20 robot-assisted gait training sessions complemented with 20 sessions of physical therapy over 4 weeks. Separate MCIDs were calculated using 5 distribution-based approaches, complemented with an anonymized survey completed by clinical professionals.

**Results:**

The MCID range for the 6minWT was 20-38 m in the ABI cohort, with subgroup ranges of 20-36 m for GMFCS I-II, 23-46 m for GMFCS III, and 24-46 m for GMFCS IV. MCIDs for the CP population were 6-23 m, with subgroup ranges of 4-28 m for GMFCS I-II, 9-19 m for GMFCS III, and 10-27 m for GMFCS IV. For GMFM-88 total score, MCID values were 1.1%-5.3% for the ABI cohort and 0.1%-3.0% for the CP population. For dimension “D” of the GMFM, MCID ranges were 2.3%-6.5% and 0.8%-5.2% for ABI and CP populations, respectively. For dimension “E,” MCID ranges were 2.8%-6.5% and 0.3%-4.9% for ABI and CP cohorts, respectively. The survey showed a large interquartile range, but the results well mimicked the distribution-based methods.

**Conclusions:**

This study identified for the first time MCID ranges for 6minWT and GMFM-88 in pediatric patients with neurological impairments, offering useful insights for clinicians to evaluate the impact of treatments. Distribution-based methods should be used with caution: methods based on pre-post correlation may underestimate MCID when applied to patients with small improvements over the treatment period. Our results should be complemented with estimates obtained using consensus- and anchor-based approaches.

## 1. Introduction

Cerebral palsy (CP) and acquired brain injury (ABI) are commonly associated with gait disorders [1, 2]. While CP is the most common cause of motor, sensory, and cognitive disability in childhood, ABI is the leading cause of mortality and lifelong disability in children. Traumatic brain injury is the most frequent cause of ABI, while nontraumatic ABI, such as stroke and tumor, have lower incidence [3]. In both CP and ABI, rehabilitation medicine and physical therapy programs play a crucial role in the multidisciplinary approach representing the gold standard of care [4, 5].

Robot-assisted gait training (RAGT) is a well-established rehabilitation tool used to improve walking ability in patients with gait deficits. RAGT systems impose a regular gait pattern while allowing a degree of body-weight support in combination with proper alignment of the lower limbs [6].

Common outcome measures that have been used to establish the effectiveness of RAGT training include measures of walking endurance such as the distance walked during the 6-minute walking test (6minWT) and gross motor function measures (GMFM-88 and GMFM-66). Some studies on children with CP have shown statistically significant improvements in 6minWT [7–9]. Most of the studies included in a recent review [10] also noted statistically significant improvements in dimensions “D” (associated to functional abilities related to standing) and “E” (associated to walking, running, and jumping) of the GMFM-88, with a slightly major effect on dimension “D.” However, evidence is still weak and inconsistent and partially depends on the treatment approach used [11]. Only very few studies have investigated the benefits of RAGT on diagnoses other than CP, especially in pediatric cohorts: significant improvements in GMFM-88 scores were highlighted in ABI patients after 20 RAGT sessions [12, 13]. However, a statistically significant change may not be perceived by the patient. The concept of the minimal clinical important difference score (MCID) was originally developed to overcome this limitation and to reflect the patient's perspective of change [14]. The MCID is a standard way of measuring clinical relevance and is increasingly being used to guide clinical decision-making and to determine the quality of an intervention [15]. In its original definition, the MCID was defined as the smallest improvement that a patient could perceive as beneficial and that determined a change in his/her management [16]. Methodologically, the use of anchor-based methods, linking the change in score obtained for a given intervention to patient-reported improvements, reflects closely the original definition of MCID, but can be affected by recall bias. On the contrary, MCIDs can also be obtained using distribution-based approaches [17], which rely solely on statistical characteristics of the sample. They account for random variations in the population, but do not consider the participant's perspective. The most common distribution-based methods currently used to establish the MCID are based on standard deviation, effect size, standard error of measurement, and standardized response mean [14]. In addition, anchor- and distribution-based approaches may be complemented with a consensus process among clinicians which may allow to narrow the range of potential MCID values [18]. The determination of MCID values using distribution-based methods in pediatric populations other than ABI and CP [19], especially those using pre-post characteristics of the study sample, has provided methodological evidence for the design of phase 2 study research protocols using the change from baseline in 6minWT distance as the primary efficacy endpoint [20].

To our knowledge, only one study has already established MCID values for outcome measures used to reflect improvements in pediatric CP populations. In their longitudinal study on 381 children with CP, Oeffinger and colleagues calculated MCID thresholds for several outcome measures for patients classified at different levels of the Gross Motor Function Classification System (GMFCS), including GMFM-66 total score and dimensions “D” and “E” of GMFM-88 [21]. MCID scores for clinical tests evaluating walking endurance, such as the 6minWT, are still lacking [22]. In addition, there are no studies that have provided MCID scores in ABI populations.

The aim of this work is to estimate and critically evaluate the MCID for the 6minWT and the GMFM-88 using a range of distribution-based approaches after a RAGT treatment in a group of pediatric patients affected by gait disorders due to ABI and CP, grouped by GMFCS levels. These values are complemented with MCIDs obtained from clinical professionals expert in rehabilitation treatments using a survey. Results provided may be used as benchmarks by clinicians to compare improvements for ambulant children with CP and ABI.

## 2. Materials and Methods

### 2.1. Participants

The present analysis includes data from a retrospective study on 182 children with clinical conditions of ABI (*n* = 110) or CP (*n* = 72) and Gross Motor Function Classification System (GMFCS) levels I-IV, whose results are published elsewhere [23]. Inclusion criteria are as follows: (1) pediatric age and adolescence (4-18 years) with diagnosis of ABI or CP; (2) ability to communicate pain, fear, or discomfort; (3) ability to walk independently with or without the use of assistive devices or orthoses; (4) cooperation for assessment; (5) minimum femur size of 23 cm for an appropriate use of RAGT; and (6) a regular routine in physiotherapy treatment before this study. Children with severe cognitive, visual, or perceptive deficit were excluded from the study. Children with cardiovascular diseases; surgery within the past 12 months; severe spasticity; or with a passive range of motion limitations at the ankle, knee, or hip level (i.e., participants who cannot match the robot joint rotations) were also excluded. This study was performed in accordance with the Declaration of Helsinki, and the protocol was approved by the IRCCS E. Medea Ethics Committee. The ethical committee stated that informed consent was not required for this retrospective observational study. The trial has been registered on ClinicalTrials.gov (NCT03828110).

### 2.2. Intervention and Outcome Measures

RAGT was performed using a commercially available driven gait orthosis (Lokomat, Hocoma AG, Volketswil, Switzerland). The rehabilitation protocol consisted in 20 sessions of RAGT complemented with 20 sessions of standard physical therapy over a period of 4 weeks. RAGT and conventional physical therapy sessions lasted 45 minutes each and were administered five times per week, during working days. Before initiating treatment (T0) and at the end of the treatment (T1), participants underwent testing by 6minWT and GMFM-88 (total score and dimensions “D” and “E”). Measures taken at baseline included age, time from trauma (for ABI patients), and GMFCS level. For a more detailed description of the rehabilitation protocol, please refer to our previously published paper [23]. Although we are not aware of any thorough validation of the GMFCS in children with ABI, it has been already used for the description of these patient population [24].

### 2.3. MCID Estimation

To estimate the MCID, five distribution-based methods were selected from recent methodological reviews reported in the literature [14, 15, 25]. The formulas were selected in order to include statistical approaches based on all the most common methods: standardized response mean, effect size, reliability, and standard deviation.

The first two methods are based on the correlation (*r*) between measures at T0 and T1 and standardized response mean (SRM) for dependent samples. The SRM is then substituted with a medium effect size of 0.5 (method M_srm) or a large effect size of 0.8 (method L_srm). The formulas are as follows [21]:

(1) $$\begin{array}{c} {\text{MCID}}_{\text{M}\_\text{srm}}=0.5*\sqrt{2}*\sqrt{\left(1-r\right)}*{\text{sd}}_{\Delta }, \end{array}$$

(2) $$\begin{array}{c} {\text{MCID}}_{\text{L}\_\text{srm}}=0.8*\sqrt{2}*\sqrt{\left(1-r\right)}*{\text{sd}}_{\Delta }, \end{array}$$

where sd_Δ_ is the standard deviation of the change score, calculated as the difference between scores at T0 and T1.

The third method (ES) is based on the assumption that the MCID is considered to be the change in score corresponding to a small effect size [26]. To calculate it, the standard deviation of the baseline scores (sd_T0_) is multiplied by 0.2 (the small effect size). The formula becomes

(3) $$\begin{array}{c} {\text{MCID}}_{\text{ES}}=0.2*{\text{sd}}_{\text{T}0}. \end{array}$$

The fourth method (SEM) uses the standard error of measurement as MCID, calculated with the following formula [27]:

(4) $$\begin{array}{c} {\text{MCID}}_{\text{SEM}}={\text{sd}}_{\text{T}0}*\sqrt{1-\text{ICC}\left[1,3\right]}, \end{array}$$

where ICC [1, 3] is the intraclass correlation coefficient. Because in our study we did not perform repeated measures to obtain ICC, values were gathered from the literature. For the 6minWT, ICC was set at 0.94 for all participants with ABI [28]. For participants with CP, ICC was set at 0.95 for the subgroup with GMFCS I-II and at 0.98 for GMFCS III and IV [29]. For the GMFM-88 total score, ICC was set at 0.99 for all ABI participants [30]. For participants with CP, ICC was set at 0.99 for the subgroups with GMFCS I-II and III and at 0.94 for GMFCS IV [31]. For the GMFM-88 dimensions “D” and “E,” ICC was set at 0.99 for both ABI [30] and CP participants [31].

The fifth method (SDB) estimates the MCID as 0.5 times the standard deviation of the change score [14]:

(5) $$\begin{array}{c} {\text{MCID}}_{\text{SDB}}=0.5*{\text{sd}}_{\Delta }. \end{array}$$

### 2.4. Survey

An online survey was prepared using REDCap electronic data capture tools hosted at IRCCS Medea [32]. The survey guaranteed anonymized responses and was administered among clinical professionals (physiotherapists and medical doctors) specialized in rehabilitation treatments. The survey was administered online by providing a link to the REDCap form, no time limit was set, and the survey had to be completed within a single session. The interviewees were provided with the mean value at baseline (T0) and the time from trauma (for ABI patients only). Based on this information, they were asked to provide MCID estimates for each of the ABI and CP subgroups, according to the definition of MCID by Jaeschke et al. [16]—“the smallest difference in score in the domain of interest which patients perceive as beneficial and which would mandate, in the absence of troublesome side effects and excessive cost, a change in the patient's management”—and their own personal judgement. They were asked to provide 6 MCID estimates (3 for ABI and 3 for CP) for each of the 4 investigated measures (6minWT, GMFM-88, GMFM DIM D, and GMFM DIM E), for a total of 24 values. In the absence of patients' reports on improvements, the information obtained from the survey was considered the reference in our study and was used to complement and compare MCIDs estimated using distribution-based techniques.

### 2.5. Analysis

The study sample was separated by diagnosis (ABI or CP) and stratified according to GMFCS level (I-II, III, and IV), similarly to previously published research [33]. The analysis estimated overall and separate MCID values for ABI and CP groups and for subgroups based on GMFCS classification.

## 3. Results

Data from 182 children with CP (*n* = 72) and ABI (*n* = 110) at GMFCS levels I-IV were analyzed. Only 152 participants completed the 6minWT both at T0 and T1. Mean age was 10.8 ± 3.9 years and 45.1% were girls. Participant details according to all subgroups are reported in Table 1.

**Figure 1:**
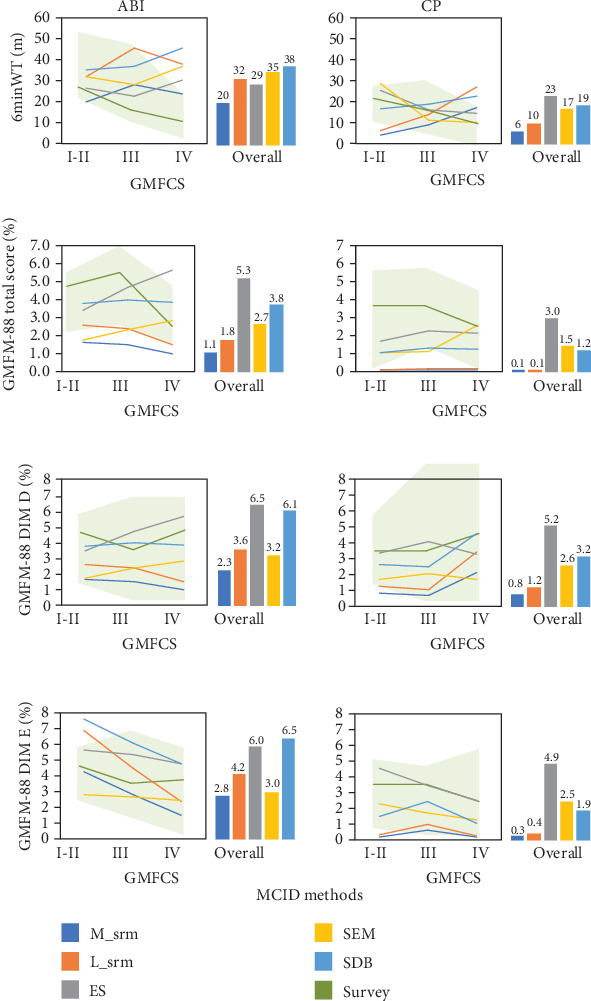
MCID estimates for the primary analysis. Graphs are organized in rows and columns, with rows corresponding to each outcome measure and columns corresponding to data from ABI and CP participants, respectively. Line graphs represent the MCID estimates for each method and for the three subgroups according to the GMFCS classification (I-II, III, and IV). The green lines represent the median MCID value obtained from the survey among expert clinical professionals, and the light green area corresponds to the survey interquartile range. The column graphs represent the overall MCID estimate. Please refer to Table 3 for all corresponding numerical values.

Scores at T0, change scores (T1-T0), and the correlation coefficient (*r*) used to estimate MCID are reported in Table 2 for all the outcomes. Estimated values of the MCID are summarized in Figure 1 and values are reported in Table 3.

For the 6minWT, absolute estimates of MCID for the ABI population ranged between 20 and 38 meters, corresponding to relative MCIDs of 9%-16%. Absolute MCIDs for subgroups classified according to GMFCS scores ranged between 20 and 36 m for GMFCS I-II, 23 and 46 m for GMFCS III, and 24 and 46 m for GMFCS IV. MCID estimates for the CP population ranged between 6 and 23 meters (corresponding to relative MCIDs of 3%-11%), with subgroup ranges of 4-28 m for GMFCS I-II, 9-19 m for GMFCS III, and 10-27 m for GMFCS IV. For GMFM-88, our study reports GMFM-88 total score MCID values ranging between 1.1% and 5.3% for the overall ABI cohort, while for the CP population the MCID range was 0.1% to 3.0%. For dimension “D” of the GMFM, MCID values for the ABI population ranged between 2.3% (M_srm) and 6.5% (ES), and between 0.8% (method M_srm) and 5.2% (method SDB) for the CP population. For dimension “E,” the present study estimated an MCID range for the ABI population of 2.8%-6.5%, while values for the CP population ranged between 0.3% and 4.9%. All values are reported in Table 3.

## 4. Discussion

The MCID score can be used to establish a priori power and sample size of a study, based on the expected effect of the therapeutic approach [34]. For this reason, defining a single MCID value for a particular outcome instrument is very attractive, but no method has been universally accepted as standard yet. While anchor-based approaches may reflect more closely the original definition of MCID, distribution-based approaches are currently widely accepted in situations where anchor-based estimates are unavailable [18]. For this reason, in this work, we aimed to estimate the MCID scores from five different distribution-based approaches in a pediatric population affected by gait disorders due to CP and ABI, and treated with RAGT. To the best of our knowledge, this is the first study providing a range of values that could serve as reference for clinicians in order to establish a therapeutic threshold for the 6minWT and the GMFM-88 for a pediatric population affected by gait disorders after one month of robotic gait rehabilitation. We selected the five most common methods with the objective of including a variety of statistical techniques on the basis of existing literature. In addition, we complemented and compared this information with MCID values suggested by a group of experienced doctors and physiotherapists, obtained using an anonymized online survey.

Despite the different statistical approaches to define the MCID, values estimated in the ABI population were higher compared to CP. This was mostly due to higher change score and baseline variability (sd_Δ_ and sd_T0_, respectively) in the ABI population. Higher expected MCIDs for patients with acquired gait impairments may reflect higher potential improvements in this group, as confirmed by higher average change scores obtained in both 6minWT and GMFM-88.

### 4.1. 6minWT

MCID values for the 6minWT have been reported in recent literature [35–37]; however, no previous study reported MCID values of the 6minWT for a pediatric population with gait disorders due to ABI or CP after a treatment period of one month, classified on the basis of their GMFCS. A recent systematic review for clinical conditions such as stroke in geriatric populations, spinal cord injuries, and chronic obstructive pulmonary disease [25] reported absolute values ranging between 13 and 45 meters for distribution-based approaches, which correspond to a relative range of 4% to 11% improvement from baseline. A study in children with Duchenne muscular dystrophy [38] reported a variable MCID range from 5.6 meters at low levels of function, to up to 46.0 meters for higher levels.

In our study, estimates of MCID for the ABI population (20-38 m) where in line with the results of Schrover et al. [25]. A similar MCID range (14.0-30.5 m) was also suggested for adults with different pathologies [37].

Absolute MCIDs for subgroups classified according to GMFCS scores did not vary significantly, suggesting that in this group of patients the minimum significant change may not strongly depend on the level of impairment. The lower MCID estimate provided by method M_srm with respect to method SDB is justified by the high T0-T1 correlations (range 0.84-0.95) used in equation (1).

Previous studies on the measurement properties of the 6minWT in CP have investigated responsiveness to change [39] and test-retest reliability [29, 40], but never reported MCID estimates in chronic pediatric conditions [35]. Lower values of the MCID in comparison to the ABI group may reflect the chronic nature of the CP population. A further consequence of this is the tendency of the methods based on variability of the change score (M_srm, L_srm, and SDB) to provide smaller MCIDs compared to the methods based on baseline variability (ES and SEM). This was especially evident for the less compromised group (GMFCS I-II), where absolute MCIDs ranged from 4 to 28 meters (for M_srm and SEM, respectively).

### 4.2. GMFM-88

The analysis of the subgroups highlighted that the MCID range is wider for patients with GMFCS IV (0.9%-5.6%) and GMFCS III (1.5%-4.6%) and narrows for patients with the lowest gait deficit (GMFCS I-II, 1.7%-3.8%): methods ES and SEM may suggest a decrease in MCID with increasing gait deficit, while methods M_srm and L_srm indicate the opposite. These results confirm that distribution-based methods may provide conflicting outcomes based on the different statistical approaches used to generate the MCID.

The very low values provided by methods M_srm and L_srm for the CP population should be interpreted cautiously, because correlations between T0 and T1 scores approached values of 1. Similarly to the values obtained for the 6minWT, lower estimates in this population compared to ABI appear to be justified by the fact that the CP population underwent less mobility improvements. The upper limit of the MCID range is comparable to data published using an anchor-based method in a group of CP patients [41], which ranged between 4.0% and 1.3%.

### 4.3. GMFM-88 Dimensions “D” and “E”

Dimension “D” of the GMFM-88 evaluates items associated to the activity of “standing.” One previous study proposed MCID estimates based on the SRM method for a CP patient cohort over a period of 1 year [21], reporting values of 1.2% (M_srm) and 1.6% (L_srm). In our study, MCIDs based on the same method were comparable although slightly lower (0.8% and 1.2% for M_srm and L_srm, respectively). The high value of MCID reported using the SDB method (5.2%) in the CP population was due to the high variability of the baseline value (sd_T0_), which may occur when nonhomogeneous subgroups are pooled together to provide an overall estimate.

Similarly, MCIDs of subgroups classified by GMFCS were lower than those reported by Oeffinger et al. and calculated using the same methods (M_srm and L_srm), while estimates computed using the ES, SEM, and SDB methods were comparable [21]. Different sample characteristics and treatment durations may have influenced the results: while change score variability reported by Oeffinger and coauthors was comparable with our data, correlations between T0 and T1 scores reported in their study were considerably higher. In addition, Oeffinger's cohort included only patients with GMFCS I, II, and III, while our population included also more severely affected patients (GMFCS IV).

Dimension “E” evaluates items associated to the activities “walking, running, and jumping.” A previous study reported an average improvement of 5% in this dimension for a similar cohort of ABI patients after RAGT rehabilitation [12]. For this population, the present study reports a trend of decreasing MCID values with increasing motor deficit. For the CP population, similarly to what was reported previously for dimension “D,” due to high T0-T1 correlations, MCIDs estimated using the M_srm and L_srm methods are lower (0.3% and 0.4%, respectively) than those previously reported (1.2% and 1.8%) for a similar cohort [21].

### 4.4. Comparison between Distribution-Based Methods

Overall, the M_srm and L_srm methods generated the smallest MCID values in our sample. The reason for this result is the high pre-post correlation associated to the 6minWT and especially to the GMFM scores. Hence, these methods should be interpreted with particular caution with values of *r* > 0.90, because they are likely underestimating the MCID. The ES method was generally associated to the largest MCID values because of its dependence on baseline variability. This method is possibly the most widely used to assess distribution-based MCID; however, some researchers have proposed different values of effect size as MCIDs [42]. The SEM and SDB methods reported overall intermediate MCIDs with respect to the previous three. The method based on standard error of the measurement has the advantage of being sample independent, but agreement on how to calculate its reliability has not yet been reached [43]. However, both methods appear appropriate to provide realistic MCID values for our cohort.

### 4.5. Survey

To date, no agreement has been found on which is the most suitable method, or “gold standard,” to obtain MCIDs. Rather, recent literature suggests triangulating between methods to obtain the most appropriate MCID value [18, 44]. In the absence of patients' reports on improvements, the information obtained using an anonymized survey administered online was considered the reference in our study and was used to complement and compare MCIDs suggested by a group of clinical professionals with values estimated using distribution-based techniques. For the 6minWT, results of the survey showed that for both CP and ABI, the expected MCID decreases as the gross motor function decreases. This trend was similar to most distribution-based methods tested, excluding those based on standardized response mean, whose increase for increasing disability was related to the increasing variability of the change score. For the GMFM total score, MCIDs from the survey appeared overall higher than those obtained using distribution-based methods, especially in the CP population, while for dimensions “D” and “E,” the MCIDs of the survey are closer to the calculated MCID values. Overall, the survey data showed large interquartile ranges. A likely reason for this is the nonuniform interpretation and estimate of the MCID among professionals: each expert clinician was asked to provide estimates for each subgroup according to the definition of MCID and his own personal judgement. In the future, a consensus-based approach may be beneficial to improve agreement between raters and provide smaller MCID ranges.

## 5. Conclusions

This study presents distribution-based estimates of MCID for the 6minWT and the GMFM-88 in pediatric gait disorders after robotic gait rehabilitation calculated using a range of methods among the most commonly reported in the literature. Data were complemented by MCIDs provided by expert clinical staff. Values provided in this study can serve as reference for clinicians in order to establish a therapeutic threshold for pediatric patients undergoing RAGT treatments. Our study confirms that caution is needed when using distribution-based methods for estimating the MCID: methods based on correlations between pre- and postscores may underestimate MCID when applied to patients with small improvements over the treatment period. However, it is promising to observe how the method based on effect size, which only relies on baseline performance of the patients, appears to well mimic the trend suggested by the survey. This method allows estimating MCID values independently from the treatment performed, giving an estimation of clinical significance while taking into account the characteristics of the sample. We suggest that research complementing distribution-based approaches with anchor-based and consensus-based MCID methods may provide a clearer picture, by linking the change in score obtained for a given intervention to patient-reported improvements.

## Figures and Tables

**Table 1:** Participant details.

	Overall	GMFCS I-II	GMFCS III	GMFCS IV
Sex (m/f)				
ABI	57/53	21/21	20/18	16/14
CP	43/29	11/12	21/10	11/7
Age (years)				
ABI	10.8 (4.1)	11.0 (3.8)	10.6 (4.0)	10.9 (4.6)
CP	10.8 (3.8)	11.0 (4.4)	11.1 (3.5)	9.9 (3.6)
Time from trauma (years)				
ABI	1.7 (1.9)	1.7 (2.2)	1.7 (1.7)	1.7 (1.9)

Mean (sd).

**Table 2:** Participant details for MCID estimation.

	T0		Change score		*r* (T0-T1)	
	ABI	CP	ABI	CP	ABI	CP
6minWT (m)						
I-II	308 (132)	319 (127)	77 (71)	23 (33)	0.70	0.89
III	192 (115)	193 (79)	73 (74)	25 (38)	0.87	0.72
IV	142 (151)	148 (73)	46 (92)	31 (45)	0.86	0.95
Overall	233 (143)	222 (117)	70 (76)	26 (38)	0.84	0.97
GMFM-88 (%)						
I-II	78.8 (17.0)	83.7 (8.3)	7.2 (7.5)	1.4 (2.0)	0.93	0.99
III	68.4 (23.2)	66.9 (11.2)	6.3 (7.9)	2.8 (2.5)	0.97	0.99
IV	43.3 (27.9)	54.1 (10.4)	7.1 (7.6)	1.4 (2.4)	0.96	0.99
Overall	65.5 (26.5)	69.1 (15.1)	6.8 (7.6)	2.0 (2.4)	0.91	0.99
DIM D (%)						
I-II	70.5 (23.5)	72.6 (16.6)	10.9 (11.7)	3.8 (5.2)	0.93	0.97
III	61.8 (28.9)	44.6 (20.3)	6.5 (10.4)	5.6 (5.0)	0.91	0.89
IV	26.7 (29.6)	23.8 (16.4)	10.3 (14.1)	4.3 (9.1)	0.93	0.97
Overall	55.5 (32.4)	48.3 (26.0)	9.2 (12.1)	4.7 (6.3)	0.88	0.96
DIM E (%)						
I-II	54.0 (28.0)	57.7 (22.5)	13.3 (15.2)	1.1 (2.9)	0.89	0.97
III	39.0 (26.5)	27.1 (17.1)	9.1 (12.1)	2.4 (4.8)	0.95	0.99
IV	17.3 (23.9)	15.1 (12.4)	7.4 (9.5)	0.9 (2.0)	0.91	0.99
Overall	38.8 (30.0)	33.8 (24.7)	10.3 (12.9)	1.6 (3.7)	0.84	0.99

Mean (sd).

**Table 3:** MCID estimates for all outcome measures for the 5 distribution-based approaches and the survey, divided for ABI and CP etiologies and GMFCS subgroups.

	M_srm		L_srm		ES		SEM		SDB		Survey	
	ABI	CP	ABI	CP	ABI	CP	ABI	CP	ABI	CP	ABI	CP
6minWT (m)												
I-II	20	4	32	6	26	25	32	28	36	17	30	25
III	29	9	46	14	23	16	28	11	37	19	20	20
IV	24	17	38	27	30	15	37	10	46	23	15	14
Overall	20	6	32	10	29	23	35	17	38	19	—	—
GMFM-88 (%)												
I-II	1.6	0.1	2.5	0.1	3.4	1.7	1.7	1.0	3.8	1.0	5.0	4.0
III	1.5	0.1	2.3	0.2	4.6	2.2	2.3	1.1	4.0	1.3	5.7	4.0
IV	0.9	0.1	1.5	0.1	5.6	2.1	2.8	2.5	3.8	1.2	3.0	3.0
Overall	1.1	0.1	1.8	0.1	5.3	3.0	2.7	1.5	3.8	1.2	—	—
DIM D (%)												
I-II	2.9	0.8	4.6	1.2	4.7	3.3	2.4	1.7	5.9	2.6	5.0	4.0
III	1.9	0.6	3.0	1.0	5.8	4.1	2.9	2.0	5.2	2.5	4.0	4.0
IV	3.0	2.1	4.8	3.4	5.9	3.3	3.0	1.6	7.1	4.6	5.1	5.0
Overall	2.3	0.8	3.6	1.2	6.5	5.2	3.2	2.6	6.1	3.2	—	—
DIM E (%)												
I-II	4.3	0.2	6.8	0.3	5.6	4.5	2.8	2.3	7.6	1.5	5.0	4.0
III	2.8	0.6	4.5	1.0	5.3	3.4	2.7	1.7	6.1	2.4	4.0	4.0
IV	1.5	0.1	2.4	0.2	4.8	2.5	2.4	1.2	4.8	1.0	4.2	3.0
Overall	2.8	0.3	4.4	0.4	6.0	4.9	3.0	2.5	6.5	1.9	—	—

## Data Availability

The authors confirm that the data supporting the findings of this study are available within the article. The complete raw data that support the findings of this study are available from the corresponding author (FA) upon reasonable request.
